# Who Is Most Vulnerable to Social Rejection? The Toxic Combination of Low Self-Esteem and Lack of Negative Emotion Differentiation on Neural Responses to Rejection

**DOI:** 10.1371/journal.pone.0090651

**Published:** 2014-03-04

**Authors:** Todd B. Kashdan, C. Nathan DeWall, Carrie L. Masten, Richard S. Pond, Caitlin Powell, David Combs, David R. Schurtz, Antonina S. Farmer

**Affiliations:** 1 Department of Psychology and Center for the Advancement of Well-Being, George Mason University, Fairfax, Virginia, United States of America; 2 Department of Psychology, University of Kentucky, Lexington, Kentucky, United States of America; 3 Center for the Mind and Brain, University of California-Davis, Davis, California, United States of America; 4 Department of Psychology, University of North Carolina Wilmington, Wilmington, North Carolina, United States of America; 5 Department of Psychology, Georgia College & State University, Milledgeville, Georgia, United States of America; Univ of Toledo, United States of America

## Abstract

People have a fundamental need to belong that, when satisfied, is associated with mental and physical well-being. The current investigation examined what happens when the need to belong is thwarted—and how individual differences in self-esteem and emotion differentiation modulate neural responses to social rejection. We hypothesized that low self-esteem would predict heightened activation in distress-related neural responses during a social rejection manipulation, but that this relationship would be moderated by negative emotion differentiation—defined as adeptness at using discrete negative emotion categories to capture one's felt experience. Combining daily diary and neuroimaging methodologies, the current study showed that low self-esteem and low negative emotion differentiation represented a toxic combination that was associated with stronger activation during social rejection (versus social inclusion) in the dorsal anterior cingulate cortex and anterior insula—two regions previously shown to index social distress. In contrast, individuals with greater negative emotion differentiation did not show stronger activation in these regions, regardless of their level of self-esteem; fitting with prior evidence that negative emotion differentiation confers equanimity in emotionally upsetting situations.

## Introduction

Humans have unique attributes, idiosyncrasies, and other qualities that set them apart from others. Some people chronically perceive that others reject or exclude them, leading them to have relatively low self-esteem [1], [2]. Other people consistently perceive that others accept and include them, leading them to have relatively high self-esteem. Despite these differences, most humans share a common desire to have a few positive and lasting relationships that, when thwarted, produces an assortment of negative consequences for mental and physical well-being [3]–[11].

Various brain regions have been implicated in the process by which people monitor whether others are evaluating them—and activation in these regions is modulated by individual differences in self-esteem [12], [13]. What remains unclear, however, is whether this modulation is affected by people's ability to be aware of and clarify/distinguish their negative emotional experiences (a defining feature of emotional intelligence [14], [15]).

The current study fills this gap in the literature by focusing on how individual differences in emotion differentiation—the ability to differentiate one's emotional experiences into discrete categories [16], [17]—may moderate the relationship between self-esteem and neural responses to social rejection. We predicted that self-esteem would modulate neural responses to social rejection, such that lower levels of self-esteem would relate to greater activation in the brain regions associated with ‘social pain’—the negative affective experience that accompanies rejection by others. Crucially, we anticipated that this modulation would be isolated to people who have deficiencies in their ability to differentiate their negative emotional experiences into discrete categories. Hence, low self-esteem, coupled with low emotion differentiation, would represent a toxic combination in terms of neural responses to social rejection.

### Neural Responses to Social Rejection and Social Feedback: Self-Esteem Matters

Neuroimaging research has identified some of the primary regions that are involved in the experience of being rejected by others. Specifically, the dorsal anterior cingulate cortex (dACC) and anterior insula are activated when people are being socially rejected compared to when they are being treated equally [6], [18], [19] and also in response to viewing rejection-related images [19]. Moreover, activity in the dACC and anterior insula are elevated among individuals who display greater distress as a result of being rejected by others [18], [20]. Thus, these two regions are often linked with the social pain of being rejected by others, overlapping with neural activation observed during physical pain [21].

Recently, researchers have examined whether self-esteem modulates these neural responses to social rejection. According to sociometer theory, self-esteem functions primarily to assist individuals in perceiving others' reactions and to alert individuals to the possibility of social rejection [1], [2] People who generally perceive that others reject or exclude them tend to have low self-esteem, whereas people who generally perceive that others accept or include them tend to have high self-esteem [1]. Put simply, a person's self-esteem level can be used as a gauge of that person's perceived inclusionary status [22], [23]. Because people with low self-esteem perceive that others tend to reject and exclude them, experiences of social rejection should produce greater activation in regions associated with the distress of social rejection. Moreover, people with low self-esteem may have enhanced sensitivity to the valence of social feedback they receive because such feedback provides them with information about their social standing.

This is precisely the case. Two recent investigations have examined the role of self-esteem in modulating neural responses to social rejection and social feedback. The first study used the same virtual ball-tossing paradigm that was employed in the current investigation [12]. In that study, people with low self-esteem showed the greatest activation in the dACC in response to social rejection (versus inclusion). Thus, low self-esteem was a risk factor for heightened neural distress responses associated with social rejection.

The second investigation used a different manipulation, in which participants had their picture taken and then received positive or negative feedback from confederates regarding their photograph [13]. In that investigation, participants with low self-esteem showed the greatest activation in the ventral anterior cingulate cortex/medial prefrontal cortex (vACC/mPFC) in response to positive feedback (versus negative feedback). These findings suggest that low self-esteem was associated with biased salience of social feedback.

On the surface, these two investigations produced opposite results—in one study [12], low self-esteem was associated with greater dACC activation in response to social rejection (vs. inclusion), whereas the other study [13] showed that low self-esteem was associated with stronger activation in the vACC/mPFC in response to positive feedback (vs. negative feedback). A large part of this discrepancy lies in differences between the ball-tossing paradigm and the social feedback paradigm. Yet, both investigations showed similarity in that responses previously documented in relation to the tasks (dACC activation in the ball-tossing paradigm; vACC/mPFC activation in the social feedback paradigm) were enhanced among people with low self-esteem people, suggesting that self-esteem may play an important role in modulating neural reactivity to information about one's relevant social standing.

The current work seeks to extend recent research using the ball-tossing paradigm showing that low self-esteem is associated with heightened neural distress to social rejection [12], investigating whether this relationship is more pronounced in people who have difficulty differentiating their negative emotional experiences into discrete categories. We focused on the ball-tossing paradigm in order to provide a first step in understanding the role of emotion differentiation on the relationship between self-esteem and neural responses to social rejection. If low self-esteem and low emotion differentiation represent a toxic combination of forces involved in neural responses to social rejection, then researchers will have a better understanding of when self-esteem matters in predicting neural responses to social rejection—and when it does not.

### Emotion Differentiation

Affective science has focused predominantly on the phenomenology of emotions. The core building blocks of emotion are valence (i.e., degree of pleasantness-unpleasantness) and arousal (i.e., the degree of energy experienced) [24], [25]. Human beings have the unique ability to describe and reflect on their feelings, often referred to as meta-emotion and meta-cognition. Commonly investigated aspects of meta-emotion include people's ability to identify, understand, differentiate, and verbally describe what is being felt at a given point of time (e.g., [26]–[28]). Specifically, a diminished ability to differentiate one's emotions predicts behavioral responses and well-being to a greater degree than simply the intensity and frequency of emotions experienced [29], [30]. Of most relevance to the current study, the process of emotional differentiation can influence how people react to stressful situations.

Most researchers interested in emotion differentiation have relied on single-occasion, cross-sectional survey designs. For instance, people scoring lower on a self-report scale of emotion differentiation recovered more slowly after being shown a distressing film, and ruminated more about the experience over time [31]. Other studies have shown that trait survey measures of emotion differentiation correlate positively with openness to experience [32] and positive social functioning [33]. However, the methodological limitations of these studies limit our ability to form inferences about the value of differentiating emotions. To understand the temporal sequencing from emotion differentiation to adaptive self-regulation, there is value in collecting within-person data over time, in the natural contexts where people experience, reflect on, and react to emotions and stressors.

In a small body of studies, instead of asking people to answer questions on a 7-point Likert scale of whether they understand and differentiate their emotions (e.g., [31], [33]), researchers measured their tendencies to classify felt experiences into discrete emotion categories across multiple situations over time [16], [17], [34]. Being unable to consistently attend to, clarify, and differentiate what is being felt in a given moment should decrease the amount of cognitive capacity that is available to process initial emotional responses to stress [35], [36]. Resulting consequences should include greater stress reactivity [37], [38]. With data from daily process approaches, where information is collected from random moments or at the end of each day for several weeks, the evidence supports these theoretical models. Specifically, people who fare worse in discerning what they feel in their daily life showed greater risk when confronting stress, and possessed greater negative attitudes and greater distress about intense emotions [17], [34], [37], [39]. In contrast, individuals who show ease at discerning what they are feeling beyond crude descriptions of “pleasant” or “unpleasant” states show less intense, short-lived distress reactions.

In the current study, we collected data on people's self-esteem and emotional experiences every day over the course of a three-week assessment period [40]. By using this rich, within-person data to assess individual differences, we can be more confident that we captured the dynamic nature of people's personality. Given their heightened risk for distress in response to upsetting events, we predicted that people with low self-esteem who were also low in emotion differentiation would show the strongest activation in the dACC and anterior insula in response to social rejection. In contrast, we predicted that self-esteem would bear no significant relationship to activation in these regions among people high in emotion differentiation. We focused on emotion differentiation of negative emotional states because they are most closely associated with social rejection [41].

## Method

### Ethics Statement

This research and consent procedure was approved by the University of Kentucky's Institutional Review Board. All participants provided written informed consent prior to participating in the study. There were no minors/children enrolled in the study. Participant consent was recorded via paper-and-pencil forms.

### Participants

Participants included 25 (16 females) healthy, right-handed undergraduates (mean age 20.94, *SD*  = 5.24). They reported no history of claustrophobia and were thoroughly screened for metal and other MRI contraindications.

Ten participants had been taking daily doses of acetaminophen (the remainder took placebo) for the three weeks preceding the scan, as part of a separate study examining effects of acetaminophen vs. placebo on neural responses to social exclusion [61]. To ensure that this pre-scan exposure to acetaminophen (or placebo) did not impact the current findings, we controlled for condition (acetaminophen vs. placebo) in all behavioral and neuroimaging analyses. Neither self-esteem nor emotion differentiation significantly differed as a function of experimental condition. There is no overlap between any of the analyses reported in the current report and those reported in DeWall et al [61].

### Procedures

Three weeks before the scan, participants completed daily records on a dedicated website about their self-esteem and negative emotional experiences over a three-week assessment period. All daily entries were time-and-date stamped, assuring that a single entry was completed each day. From this within-person data, we assessed dispositional self-esteem and emotion differentiation (see details below). On the day of the scan, participants were told they would play a virtual ball-tossing game in the scanner (Cyberball [8]), which would be played via the Internet with two other same-sex participants in other scanners. To enhance the credibility of the task, participants were provided with personal information about the other players (e.g., name, age, hometown, major area of study) so that they could become ‘acquainted’ with them before playing the ball-tossing game. In reality, participants played with a preset computer program, and the player information was prepared in advance.

At the beginning of each round of the game, two virtual players appeared in the top left and right corners of the computer screen. An arm was located at the bottom center of the screen, which represented the participant's hand. After 9 seconds, the virtual player located in the top left corner began the game by tossing the ball to one of the players. Each time participants received a ball toss, they indicated which of the other players they would like to toss the ball to next by pressing one of two buttons. In the first round of the game, participants were included for the entire duration of the game. In the second round, the other players stopped throwing the ball to the participant after he/she had received three throws. Participants were excluded for the remainder of the game and watched while the other players continued the game without them. Following the scan, participants reported their social distress resulting from this exclusion (see details below), in order to ensure that participants noticed the exclusion and felt distress as a result. Finally, they were debriefed about the deception involved in the study and were given an opportunity to withdraw their data. No participant expressed suspicion regarding the cover story or chose to withdraw their data.

### Behavioral Measures

#### Self-esteem

At the end of each day for a three-week assessment period, participants completed the state self-esteem scale (SSES [42]). The SSES assesses fluctuations in feelings of self-worth across three dimensions: social, performance, and appearance. This measure consists of 20 items, which are each answered using a 5-point scale from 1 (*not at all*) to 5 (*extremely*). Example items include “I am worried about whether I am regarded as a success or a failure” (social subscale; reverse-scored), “I feel confident about my abilities” (performance subscale), and “I feel satisfied about the way my body looks right now” (appearance subscale). Items were reverse-coded when appropriate and summed for each day to create a daily composite score for self-esteem across all three dimensions.

Using the program Hierarchical Linear Modeling 6.08 [43], we examined the reliability of this daily diary measure with a three-level unconditional model with items nested within days, and days nested within people (see [44] for rationale). In such an analysis, the reliability of the Level 1 intercept is the functional equivalent of a day level Cronbach's alpha, adjusted for differences among days and among people. Results found that the 20 items of daily self-esteem were reliable (.98). Prior work has shown that the SSES shows consistently high correlations with trait self-esteem measures and has a similar relationship in predicting neural responses to social evaluative feedback [13]. Therefore, we focused our analyses on total SSES scores over three weeks to provide a valid assessment of individual differences in self-esteem.

#### Negative emotion differentiation

At the end of each day for a three-week assessment period, participants completed the negative affect subscale of the positive and negative affect schedule (PANAS [45]). The PANAS negative affect subscale assesses daily negative emotional states. The PANAS negative affect subscale consists of 10 items, which are each answered using a 5-point scale from 1 (*very slightly or not at all*) to 5 (*extremely*). Example items include “distressed”, “nervous”, and “hostile”. We examined the reliability of the daily diary measure of negative affect, again, with a three-level unconditional model with items nested within days, and days nested within people [44]. Results found that the 10 items of daily negative affect were reliable (.74).

An index of negative emotion differentiation was computed by calculating average intraclass correlations with absolute agreement between the negative emotion adjectives across the assessment period for each participant [17], [34]. Larger correlation scores indicated less differentiation of emotions. To facilitate interpretation of analyses, we then reverse coded this variable. Thus, higher values (i.e., negative correlation values that were closest to zero) pertained to higher levels of emotion differentiation.

#### Social distress manipulation check

Immediately following the scan, participants completed the Need-Threat Scale (NTS [8], [46]), which measures social distress resulting from the exclusion round of the game. The NTS assesses 20 subjectively experienced consequences of being excluded, including ratings of: self-esteem (“Playing the game made me feel insecure”), belongingness (“I felt like an outsider during the game”), meaningfulness (“I think it was useless that I participated in the game”), and control (“I had the feeling that I affected the course of the game”), using a scale ranging from 1 (*strongly disagree*) to 7 (*strongly agree*). Items were reverse coded when appropriate and averaged to create a composite score of social distress with high reliability (α = .92). In the present study, descriptive information for the NTS is detailed as part of the results section as evidence that participants were aware of, and distressed by, the exclusion during Cyberball. Correlations between NTS scores and brain activity during exclusion compared to inclusion (examined via both ROI and whole brain analyses) for this sample of participants are reported elsewhere [47].

### fMRI Data Acquisition

Data were aquired on a 3 Tesla Siemens Trio scanner at the University of Kentucky. Functional neuroimaging data were collected during each round of the ball-tossing game using a T2*-weighted gradient echo sequence with the following parameters: 30 ms echo time, 64×64 matrix, 224×224 mm field of view, 40 3.5-mm axial, slices acquired in interleaved order, 2 s repetition time. These parameters allow whole brain coverage with 3.5 mm cubic voxels. A 3D shim was performed before all EPI image acquisitions.

### fMRI Data Analysis

Neuroimaging data were preprocessed and analyzed using Statistical Parametric Mapping (SPM5; Wellcome Department of Cognitive Neurology, Institute of Neurology, London, UK). Preprocessing of the neuroimaging data included realignment of images to correct for head motion, normalization of images into a standard stereotactic space defined by the Montreal Neurological Institute and the International Consortium for Brain Mapping, and spatial smoothing using an 8 mm Gaussian kernel, full width at half maximum, to increase signal-to-noise ratio.

Each round of the game was modeled as a run with each period of inclusion and exclusion modeled as blocks within the run for a total of two inclusion blocks (one during the first run (60 seconds) and one during the second run prior to exclusion (42 seconds) and one exclusion block (60 seconds). The order of the runs was kept constant across all participants. After modeling the ball-tossing game, we calculated linear contrasts for each participant comparing the exclusion block to the inclusion blocks. These individual contrast images were then used in ROI analyses across all participants.

### Region of Interest Analyses

Based on a priori hypotheses regarding the involvement of the dACC and anterior insula in processing social rejection, we utilized anatomically-defined region of interest (ROI) analyses. Thus, we calculated differential activity in each ROI during exclusion versus inclusion, and examined how this activity related to individuals' self-esteem, emotion differentiation, and the self-esteem by emotion differentiation interaction (significance was defined as *p*<.05).

**Figure 1:**
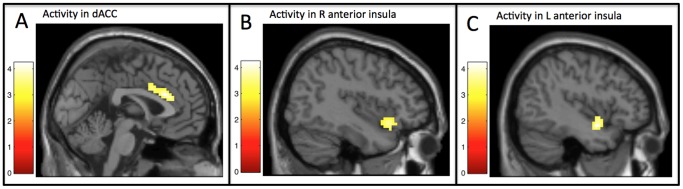
Regions of interest for exclusion vs. inclusion analyses. Regions of the dACC (panel A; [0 24 24]), right anterior insula (panel B; [39 21–15]), and left anterior insula (panel C; [−39 0 −15]) in which activity during exclusion vs. inclusion related to the interaction between self-esteem and emotion differentiation (after controlling for acetaminophen condition; see Footnote 1).

ROI extraction was performed using the Marsbar toolbox within SPM. The dACC ROI was anatomically defined as the portion of Brodmann's areas 24 and 32 (as defined by the PickAtlas) posterior to *y* = 34. It was defined as a single midline structure, rather than two separate right and left regions, given the lack of spatial separation between its right and left hemispheric portions and to be consistent with standard anatomical definitions (see Figure 1, Panel A). The bilateral anterior insula ROI was anatomically defined as the portion of the insula, as defined by the AAL atlas that is located anterior to *y* = 0 (see Figure 1, Panels B and C). Mean parameter estimates for each participant (that model the amplitude of the BOLD response during exclusion vs. inclusion) were extracted and averaged across all the voxels in each ROI. Details of the main effect analyses testing this difference in activity during exclusion compared to inclusion within each ROI are reported elsewhere [47].

To examine the interactive effect of self-esteem and emotion differentiation related to heightened activity during exclusion versus inclusion in each ROI, these parameter estimates were entered as dependent variables in multiple regression analyses in SPSS. Specifically, for each ROI a hierarchical regression analysis was performed in which self-esteem and emotion differentiation were entered in the first step and the self-esteem by emotion differentiation interaction was entered in the second step (following the guidelines of [48]). We also controlled for the effect of condition by including it as a covariate in all analyses (see Footnote 1). Thus, we examined the role of emotion differentiation in moderating the impact of self-esteem on neural activity during exclusion versus inclusion in the dACC and anterior insula ROIs. Given our directional predictions, all significance tests were one-tailed.

## Results

### Descriptive Information

SSES scores ranged from 47.89 to 95.19 (*M* = 76.29, *SD*  = 13.11), which is consistent with previously published scores [13], [42]. Emotion differentiation scores ranged from −0.03 to 0.91 (*M* = 0.52, *SD*  = 0.28).

### Social Distress Manipulation Check

Participants reported moderate levels of social distress following the exclusion round of Cyberball, with NTS scores ranging from 2.10 to 5.80 (*M* = 3.99, *SD*  = 1.00). Following Bolling and colleagues [49], we conducted a one-sample t-test to determine whether NTS scores differed significantly from the minimum score of 1 that would reflect no social distress. As expected, the average NTS score was significantly different than the minimum score of 1, *t* = 19.66, *p*<.001. This suggests that the Cyberball manipulation was successful in eliciting rejection-related feelings of distress among participants.

### Region of Interest Regression Analyses

We examined how self-esteem and emotion differentiation interacted to predict the difference in activity during exclusion versus inclusion in the dACC and anterior insula ROIs. We conducted a hierarchical regression analysis in which self-esteem and emotion differentiation were entered in the first step and the self-esteem by emotion differentiation interaction was entered in the second step. To facilitate interpretation, we centered all predictors prior to analysis.

**Figure 2:**
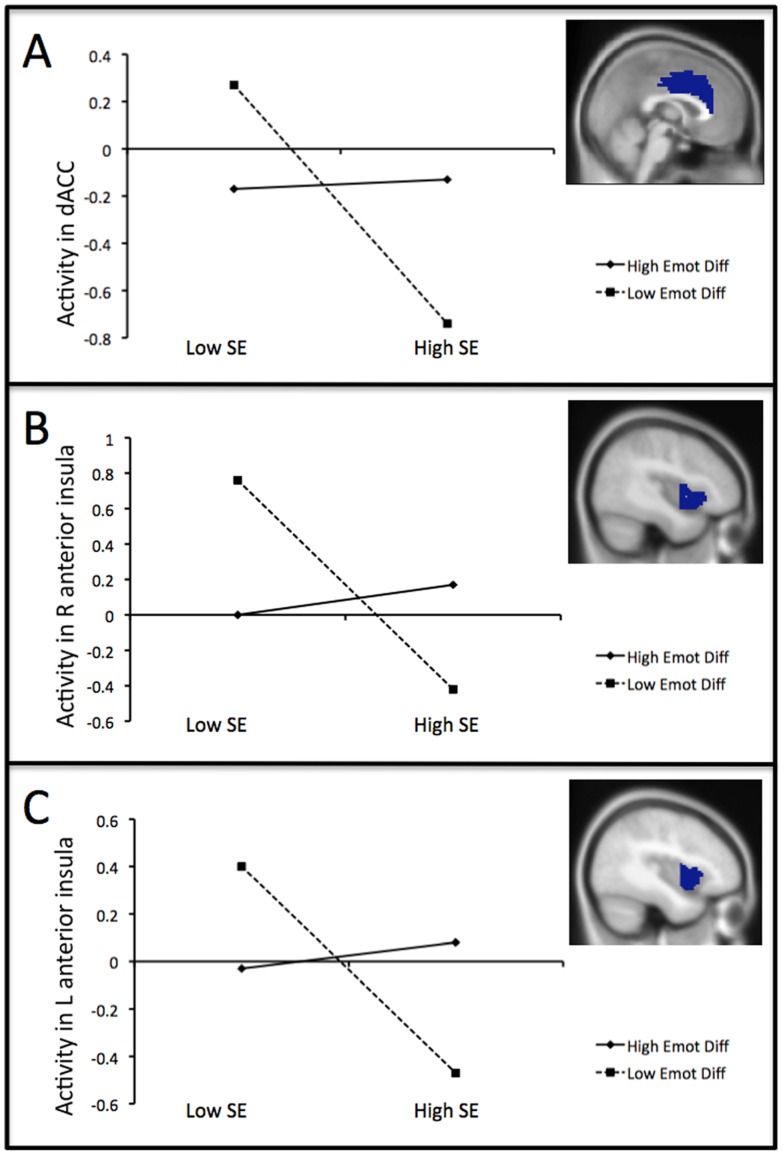
Interactive effects of emotion differentiation and self-esteem on neural activation during exclusion vs. inclusion. Associations between self-esteem (SE) and activity during exclusion vs. inclusion in the dACC (panel A), right anterior insula (panel B), and left anterior insula (panel C) ROIs, shown separately for high emotion differentiators and low emotion differentiators.

As expected, there was a significant self-esteem by emotion differentiation interaction in the dACC ROI, *β* = 0.41, *t* = 2.24, *p* = .02 (see Figure 2, Panel A). The main effect for self-esteem was also significant, which replicates prior working showing that low self-esteem is associated with greater cingulate activation to social rejection, *β* = −0.43, *t* = −1.98, *p = *.03. The emotion differentiation main effect did not approach significance, *β* = 0.08, *t* = 0.36, *p* = .36. To clarify the nature of the interaction, we examined the effect of self-esteem at relatively low (i.e., 1 standard deviation below the mean) and high (i.e., 1 standard deviation above the mean) levels of emotion differentiation [48]. Among low emotion differentiators, lower self-esteem was strongly associated with greater dACC activation in response to social rejection, *β* = −0.90, *t* = −3.70, *p*<.001. In contrast, self-esteem bore no significant relation to dACC activation among high emotion differentiators, *β* = 0.04, *t* = 0.12, *p = *.45.

Next, we re-ran these analyses using separate ROIs in the right and left anterior insula. The self-esteem by emotion differentiation interaction was significant for both the right (*β* = 0.46, *t* = 2.68, *p*<.01; see Figure 2, Panel B) and left (*β* = 0.43, *t* = 2.28, *p* = .02; see Figure 2, Panel C) anterior insula. Main effects for self-esteem [right: *β* = −0.40, *t* = −1.95, *p* = .03; left: *β* = −0.38, *t* = −1.68, *p* = .05] and emotion differentiation [right: *β* = −0.07, *t* = −0.34, *p* = .37, left: *β* = 0.06, *t* = 0.27, *p* = .40] indicated that lower self-esteem predicted greater anterior insula activation and emotion differentiation was unrelated to anterior insula activation.

Among low emotion differentiators, lower self-esteem predicted higher right (*β* = −0.92, *t* = −4.05, *p*<.001) and left (*β* = −0.87, *t* = −3.47, *p* = .001) anterior insula activation in response to social rejection, whereas high emotion differentiators showed no significant activation in the right (*β* = 0.13, *t* = 0.39, *p* = .35) or left (*β* = 0.12, *t* = 0.32, *p* = .38) anterior insula. Thus, low self-esteem and low emotion differentiation provided a negative combination in predicting neural responses to social exclusion. People high in emotion differentiation consistently reported greater equanimity, with similar reactivity to the inclusion and exclusion conditions in both the dACC and anterior insula.

## Discussion

Most people want to be part of a human pack. In our evolutionary history, humans lived in small groups in which social rejection caused more than tears and heartbreak—it often resulted in death. Because fitness is enhanced by adaptations that intensify the motivation to have positive and lasting relationships with others, humans with the neural resources to better monitor whether others are rejecting or accepting them would have optimized their ability to survive and reproduce. This capacity to gauge one's inclusionary status forms the basis for why people have self-esteem [1], [2] People who perceive themselves as rejected from the pack tend to have low self-esteem, and they show enhanced sensitivity to feedback regarding their social standing [12], [13]. Nevertheless, low self-esteem may modulate neural activation to social rejection depending on how well people identify, understand, and differentiate their negative emotional experiences. Given prior work demonstrating that low emotion differentiation is associated with heightened distress to upsetting events [17], [37], [39] we predicted that low self-esteem and low emotion differentiation would prove a negative combination in predicting neural responses to social rejection. We sought to add to existing literature that studies combinations of theoretically relevant individual difference variables, instead of single constructs in isolation, to understand how people's behavior in daily life affects reactivity to social stressors.

The current study provided consistent evidence in support of the hypothesis that emotion differentiation—one of several facets of emotional intelligence [15], [50]—amplifies the risk associated with low self-esteem. In regions previously associated with responses to social rejection, lower self-esteem was associated with greater activation in regions previously associated with responses to social rejection using this paradigm (i.e., dACC and anterior insula [6], [12], [18]). Crucially, this relationship between self-esteem and neural activity was limited to participants low in emotion differentiation. Among participants high in emotion differentiation, self-esteem was unrelated to activation in these brain regions. Thus, our findings offer novel evidence regarding how individual differences in self-esteem and emotion differentiation interact to predict neural activation to social rejection.

The current work has broad implications for both fMRI and behavioral studies that investigate the role of self-esteem in predicting emotional, cognitive, behavioral, and neural responses. Although self-esteem research has flourished in the behavioral literature for several decades (see [51] for a review), relatively little research has examined the role of self-esteem in modulating neural activation. Together with other recent reports [12], [13], the current research suggests that neuroscientists can profit from exploring the implications of individual differences in self-esteem for drawing *functional inferences* about brain systems and *psychological inferences* about the mechanisms underlying behavior [52]. Crucially, the current work suggests that emotion differentiation can accentuate or eliminate the relationship between self-esteem and neural activation. To our knowledge, this is the first report to demonstrate that emotion differentiation interacts with self-esteem to predict any type of response. Self-esteem is associated with a broad array of negative outcomes (e.g., mental illness, substance dependence [53]–[55], but it is possible that these relationships are most pronounced among low emotion differentiators and absent among high emotion differentiators.

### Limitations and Future Directions

The current research offers novel evidence that neural responses to social rejection depend in part on the interaction between self-esteem and emotion differentiation. There are some limitations to the current study that may serve as avenues for future research. First, the current study did not examine whether the interactive effect of self-esteem and emotion differentiation on neural responses had implications for behavior. Social rejection impairs self-regulation [3], [5] and increases derogation of the people doing the rejecting [56]. It is possible that this pattern would be more pronounced among people who are low in both self-esteem and emotion differentiation. Future research may explore this possibility.

A second limitation is that the current study did not assess whether our effects had implications for physiological markers linked to heightened distress, such as heightened cortisol [56], [57] and proinflammatory cytokine activity [58]. Low self-esteem and low emotion differentiation may prove an especially toxic combination when predicting cortisol and proinflammatory cytokine activity to social rejection and other stressors. Moreover, recent work has shown that activation in the dACC and anterior insula to social rejection is most pronounced among people who have a strong proinflammatory cytokine response to a social stressor [59]. Future work may explore whether these effects are exacerbated among people with low self-esteem and low emotion differentiation and whether they are diminished or even eliminated among people high in emotion differentiation.

Future research may explore the role of joint attention in further modulating the interactive relationship between emotion differentiation and self-esteem on neural responses to social rejection. In a recent study, participants who responded to a joint attention bid from an animated character showed stronger activation in the anterior mPFC, whereas they showed greater activation in the ventral striatum when they initiated joint attention with an animated character [60]. By associating others with reward, having participants initiate joint attention with others may reduce the negative effects of low self-esteem and low emotion difference on neural responses to social rejection.

### Concluding Remarks

People have a fundamental need to belong that, when thwarted, produces an assortment of negative consequences. But responses to rejection are hardly uniform. People with low self-esteem respond strongly to social rejection in terms of their neural activation in regions associated with distress [12]. Our findings suggest that low self-esteem and low emotion differentiation represent a toxic combination when predicting neural responses to social rejection. From a slightly different perspective, the current work also provides evidence that an important facet of emotional intelligence—emotion differentiation—can buffer people from the pain of social rejection. Regardless of their self-esteem, high emotion differentiators showed relative equanimity in response to social rejection. By recognizing the role of emotion differentiation in shaping neural responses to social rejection, researchers will better understand when low self-esteem might be particularly problematic for responding to social rejection. Furthermore, these findings open potential clinical avenues to explore for buffering people with low self-esteem against the impact of rejection with interventions that improve emotion differentiation ability.
